# Variation of the cochlear anatomy and cochlea duct length: analysis with a new tablet-based software

**DOI:** 10.1007/s00405-021-06889-0

**Published:** 2021-05-29

**Authors:** Jennifer L. Spiegel, Daniel Polterauer, John-Martin Hempel, Martin Canis, Judith E. Spiro, Joachim Müller

**Affiliations:** 1 Department of Otorhinolaryngology, LMU Klinikum, Ludwig-Maximilians-Universität München, Marchioninistr. 15, 81377 Munich, Germany; 2 Department of Radiology, University Hospital, LMU Munich, Munich, Germany

**Keywords:** OTOPLAN, Morphology of the cochlea, CDL, Cochlear duct length, Digitalization, Anatomy of the cochlea

## Abstract

**Purpose:**

In cochlear implantation, thorough preoperative planning together with measurement of the cochlear duct length (CDL) assists in choosing the correct electrode length. For measuring the CDL, different techniques have been introduced in the past century along with the then available technology. A tablet-based software offers an easy and intuitive way to visualize and analyze the anatomy of the temporal bone, its proportions and measure the CDL. Therefore, we investigated the calculation technique of the CDL via a tablet-based software on our own cohort retrospectively.

**Methods:**

One hundred and eight preoperative computed tomography scans of the temporal bone (slice thickness < 0.7 mm) of already implanted FLEX28™ and FLEXSOFT™ patients were found eligible for analysis with the OTOPLAN software. Measurements were performed by two trained investigators independently. CDL, angular insertion depth (AID), and cochlear coverage were calculated and compared between groups of electrode types, sex, sides, and age.

**Results:**

Mean CDL was 36.2 ± 1.8 mm with significant differences between sex (female: 35.8 ± 0.3 mm; male: 36.5 ± 0.2 mm; *p* = 0.037), but none concerning side or age. Differences in mean AID (FLEX28: 525.4 ± 46.4°; FLEXSOFT: 615.4 ± 47.6°), and cochlear coverage (FLEX28: 63.9 ± 5.6%; FLEXSOFT: 75.8 ± 4.3%) were significant (*p* < 0.001).

**Conclusion:**

A broad range of CDL was observed with significant larger values in male, but no significant differences concerning side or age. Almost every cochlea was measured longer than 31.0 mm. Preoperative assessment aids in prevention of complications (incomplete insertion, kinking, tipfoldover), attempt of atraumatic insertion, and addressing individual necessities (hearing preservation, cochlear malformation). The preferred AID of 720° (two turns of the cochlea) was never reached, opening the discussion for the requirement of longer CI-electrodes versus a debatable audiological benefit for the patient in his/her everyday life.

**Supplementary Information:**

The online version contains supplementary material available at 10.1007/s00405-021-06889-0.

## Introduction

Detailed knowledge of the morphology of the cochlea has always been in the center of interest of otologists in particular prior to performing surgery. Attention has always been paid to surgical anatomy, either due to the presence of inner ear malformations [1], or the size of the cochlea and cochlear duct length (CDL) [2–4]. In addition, surgical techniques with the aim of hearing preservation [5], or the prevention of postoperative vertigo in terms of treating patients with an large vestibular aqueduct or prevention of accidental penetration of the scala vestibuli [6]. Thus, to account those issues, a variety of cochlear implant (CI) electrodes have been developed, which are offered to surgeons with different lengths, and position within the scala tympani, resulting in electrodes that are placed alongside to the lateral wall (https://s3.medel.com/pdf/21617.pdf. Accessed 8 April 2020) [7], within the middle of the scala (https://www.advancedbionics.com/content/advancedbionics/com/en/home/products/hi-focus-electrode-family.html. Accessed 8 April 2020) [8, 9], or against or close to the modiolus (http://www.cochlear.com/wps/wcm/connect/us/home/treatment-options-for-hearing-loss/cochlear-implants. Accessed 8 April 2020) [10]. Depending on the indication, the surgeon selects the appropriate electrode for each patient individually. Nowadays, thorough preoperative planning with measurement of the CDL assists in choosing the correct electrode length.

For measuring the CDL, different techniques have been introduced in the past century along with the then available technology [11]. In the mid to late nineteenth century, several researchers throughout Europe, like Retzius, Hardy, or Bredberg, used the direct method to measure the length of the cochlea on histological sections by a micrometer [3]. Until the late 1980s only two methods, direct and indirect technique, were used to measure the CDL, with the indirect method exhibiting a possible underestimation of the CDL [12, 13]. The direct technique measured the CDL directly with histologic slices, and the indirect reconstructed a two-dimensional model via a stack of histologic slices. Along with the emergence of new available technology, a computer-generated 3D model was introduced using histologic sections by Takagi and Sando [14]. In 1998, computed tomography (CT) has evolved as a useful tool to detect inner ear malformations and for preoperative CI planning. The cochlear spiral length was calculated by different dimensions of the cochlea (apical diameter and spiral constant) via an Archimedean spiral equation [15]. In 2012, after further milestone improvements by Escudé and collaborators [16], Alexiades et al. generated a formula calculating the CDL developing a more straightforward technique. Based on Escudé’s spiral equation, a linear equation was developed [17], which is also used for measurement of the average electrode radius. Using the method of Alexiades et al., the tablet-based software OTOPLAN (https://www.cascination.com/products/otoplan. Accessed 8 April 2020) [18]*,* developed by CAScination AG (Bern, Switzerland), offers an easy and intuitive way to analyze the anatomy of the temporal bone, its proportions, and to measure the CDL [19]. Therefore, we used this straightforward tablet-based software to retrospectively measure and analyze the CDL on our own cohort. Objectives of the study were to evaluate the range of CDL, find differences in different patient groups (sex, age, type of electrode), and to assess the angular insertion depth (AID) for the cochlear coverage.

## Materials and methods

### Ethical standards and patients

**Fig. 1:**
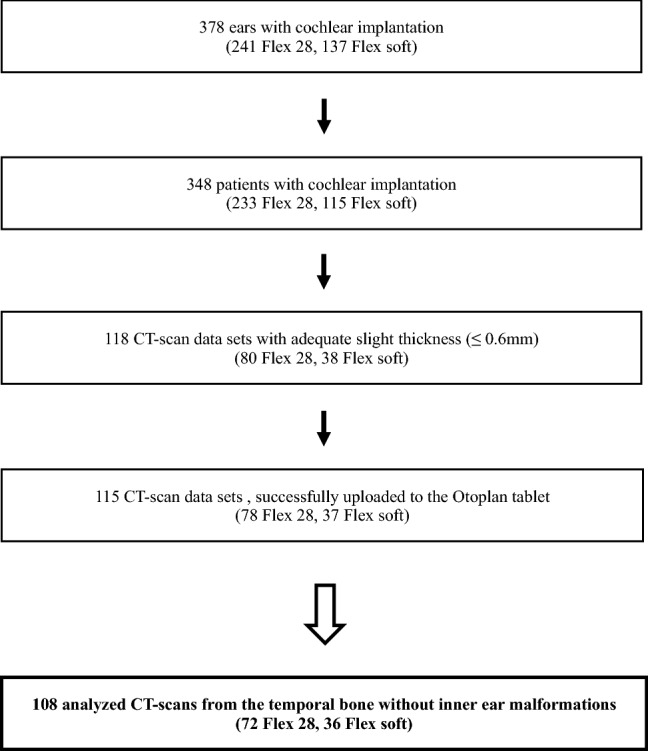
Inclusion criteria. Flowchart of cases included in and excluded from the analysis. Exclusion criteria: inner ear malformations, slice thickness ≥ 0.7 mm

We performed a retrospective analysis of preoperative CT images of 357 consecutive patients/378 consecutive ears that had undergone cochlear implantation with either a FLEX28™ or FLEXSOFT™ electrode by MED-EL (MED-EL GmbH, Innsbruck, Austria). Implantation of all patients was performed prior to availability of the tablet-based software OTOPLAN. All patients were treated between 2011 and 2018 at one academic tertiary referral center. Of those 378 CT images (radiologic Digital Imaging and Communications in Medicine (DICOM^®^), with a range of slice thickness of 0.3 to 2.0 mm, all scans were checked for eligibility of upload to the tablet. From our own experiences, a slice thickness of  ≥ 0.7 mm lead to impreciseness of measurements. Exclusion criteria were: cochlear malformations, thickness of CT slices ≥ 0.7 mm, data sets, which failed to be uploaded to the OTOPLAN software (see Fig. 1). A chart analysis of the included patients was performed after identifying candidates with the described patient’s characteristics from the CI-patient registry. Implantation of CI was performed prior to availability of the tablet-based software. FLEX 28 electrodes were implanted in patients, in whom hearing preservation was aimed. In all patients a complete insertion was achieved. All patients received postoperative standard radiologic checkup via a Stenvers view head X-ray to rule out a kinking or tip fold over of the inserted electrode, and suggested an insertion of approximately 2 turns of the cochlea equal to 720°.

The study was approved by the Institutional Review Board and Ethics Committee of the Ludwig-Maximilians-Universität München, Munich, Germany (Ethikkommission der LMU München), reference number 19-562. Procedures were followed in accordance with the ethical standards of the Helsinki Declaration [20].

### Software

The software OTOPLAN was developed by CAScination AG (Bern, Switzerland). OTOPLAN is an easy to use tablet-based software, which provides functionalities and detailed information that exceeds the usual DICOM^®^ viewer output. It features a plug and play data management, intuitive touch controls, fast and easy 3D visualization, measurement of crucial structures like the cochlea, electrode visualization, and bespoke matching as well as comprehensive patient reporting and post-op analysis. In cooperation with MED-EL it is used for preoperative planning prior to cochlear implantation. For our analysis the OTOPLAN version 2.0 (https://www.cascination.com/products/otoplan. Accessed 8 April 2020) [18] was used (CE-certification number: G1 17 10 95657 003).

### Data analysis

**Fig. 2:**
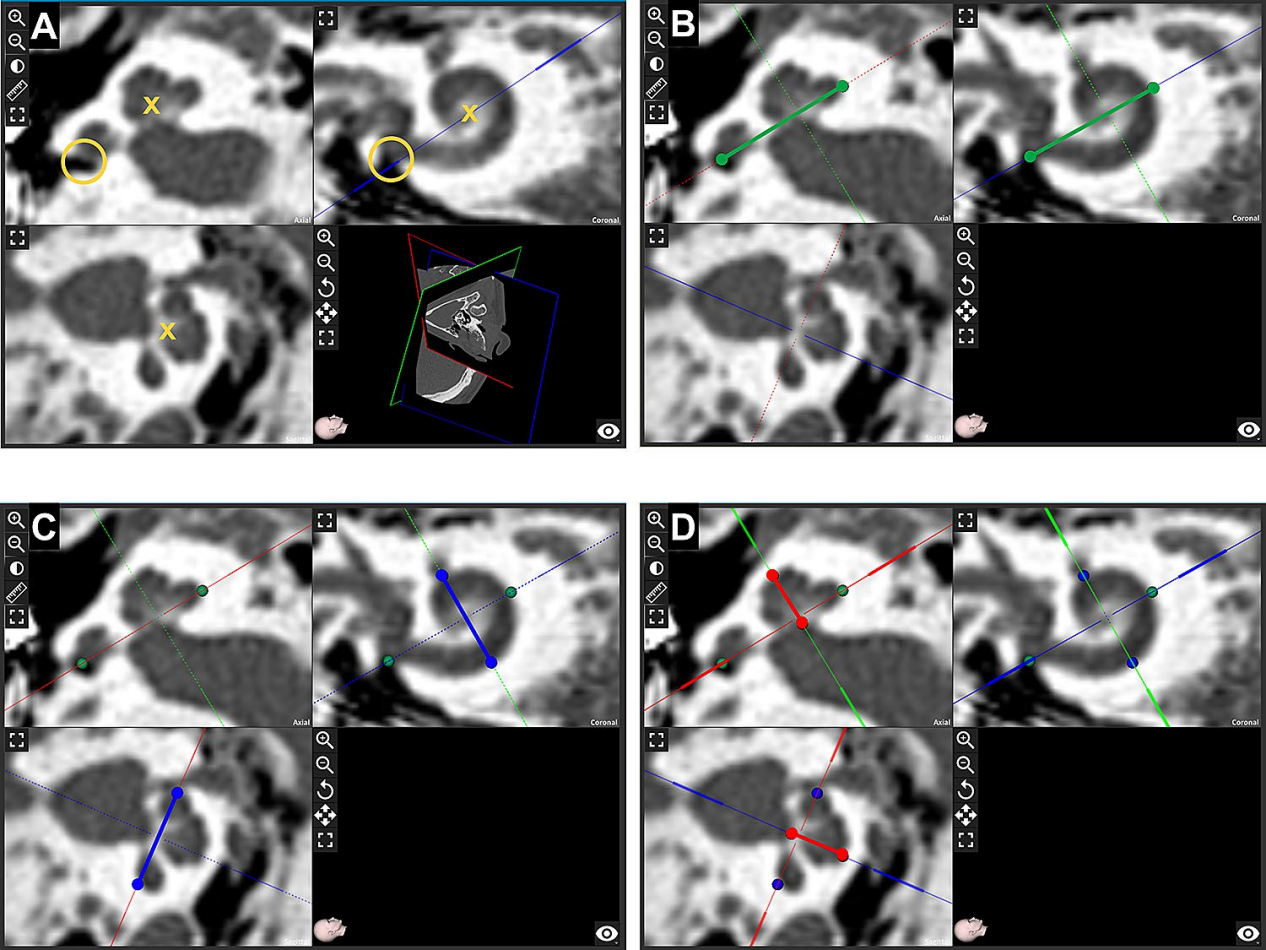
Steps of OTOPLAN. **A** Multiplanar reformation of the right inner ear reconstructed along the basal cochlear turn. The center of the modiolus (yellow cross) and the round window (yellow empty circle) are visible. **B** A value is measured as the distance between the round window and the contralateral cochlear wall: solid green line connecting two green dots. **C** B value represents the cochlear width perpendicular to the A value measurement: solid blue line connecting two blue dots. **D** Height of the cochlear is measured on a plane orthogonal to the basal turn of the cochlea: solid red line connecting two red dots

The CT scans were preoperative images, which were analyzed retrospectively after cochlear implantation. All DICOM^®^ data sets were checked for image quality and malformations of the temporal bone by a radiologist with 6 years of experience in otorhinolaryngologic imaging (J.E.S.). The remaining DICOM^®^ data were independently analyzed by two blinded investigators using multiplanar reformation (MPR): an image plane parallel to the basal turn of the cochlea was reconstructed, where ‘A value’ (largest distance from the round window to the contralateral wall) and ‘B value’ (distance between cochlear walls perpendicular to the A value line) were measured. The height of the cochlea was measured on an orthogonal plane. These details of the measurement process are illustrated in Fig. 2. With the determined values (“A”, “B”, height), the software OTOPLAN calculates the cochlear duct length according to the elliptic-circular approximation (ECA) method, which exhibits an improved accuracy compared to other techniques [21]. Thus, the cochlear coverage with AID, and cochlear place frequency on basis of the Greenwood function were calculated by the software according to a selected electrode [22]. Active stimulating length was defined by the deepest inserted point of the electrode. The FLEX28™ has a total length of 28 mm with an active stimulating length of 23.1 mm. Concerning the FLEXSOFT™, its whole length exhibits 31.0 mm and active stimulating length 26.4 mm [7]. All measurements were performed by two raters (J.E.S: radiologist; J.L.S.: otorhinolaryngologist), who were blinded to both the implanted electrodes and other rater’s results.

### Statistical analysis

The data analysis was generated using the Real Statistics Resource Pack software (Release 6.8) (Zaiontz C (2020) Real statistics using excel. www.real-statistics.com. Accessed 24 April 2020) [23] and Microsoft^®^ Excel^®^ 16.0.12730.20144. Prior to comparison of the groups, the d’Agostino-Pearson test assessed no normal distribution. Levene’s test was performed to investigate for equality of variances. Regarding comparing analysis, the Chi-square test and the Wilcoxon-Mann–Whitney *U* test was applied. Differences were considered significant at *p *values less than 0.05.

All figures were created with Microsoft^®^ Excel^®^ version 16.16.5 for iOS.

## Results

After applying exclusion criteria, out of the 378 ears, a total of 108 CT scans of the temporal bone (108 patients) were found eligible for the present study. Due to a high rate of scans with slice thickness of 0.7 mm or more, only a total of 72 FLEX28™ implanted ears and 36 FLEXSOFT™ implanted ears were included into the investigation (Fig. 1). The cohort consisted of 55 female patients (50.9%) and in 59.3% (*n* = 64) of the whole cohort the right temporal bone was analyzed. The age ranged from 6.5 to 90.3 years with a mean of 56.3 ± 19.9 years.

**Fig. 3:**
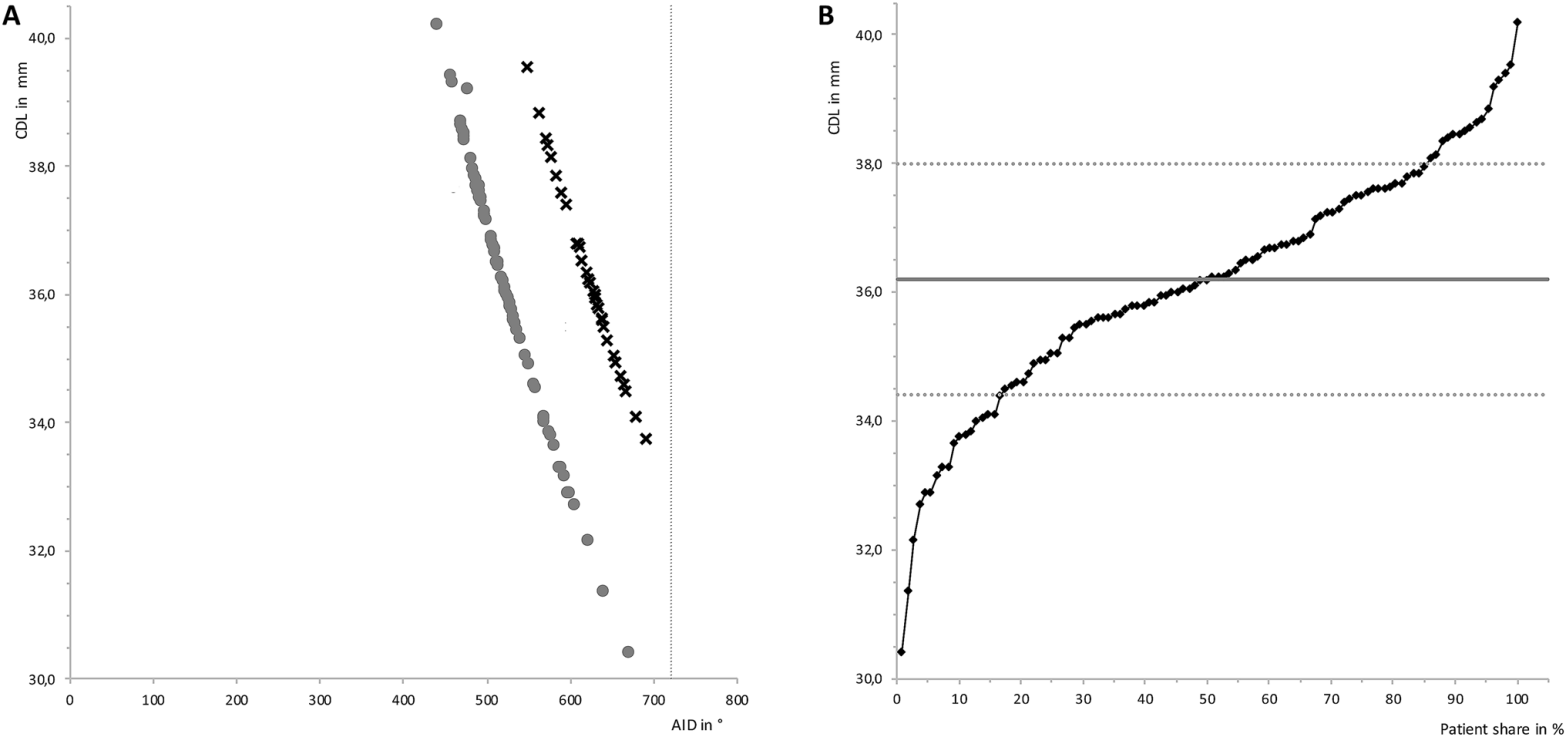
Cochlear duct length versus angular insertion depth. **A** Scatter gram of the CDL (y-axis) and cochlear coverage (x-axis) between the two electrode types FLEX28™ and FLEXSOFT™. The grey dots represent patients who received a FLEX28™ electrode, the black crosses patients with FLEXSOFT™ electrodes. The dotted grey line at the active stimulating length of 720° indicates the length of 2 windings of the cochlear. **B** Distribution function of CDL (y-axis) and share of patients in percent (x-axis). The solid grey line indicates the mean of CDL, the dotted grey line the standard deviation. *AID* angular insertion depth; *CDL* cochlear duct length

**Fig. 4:**
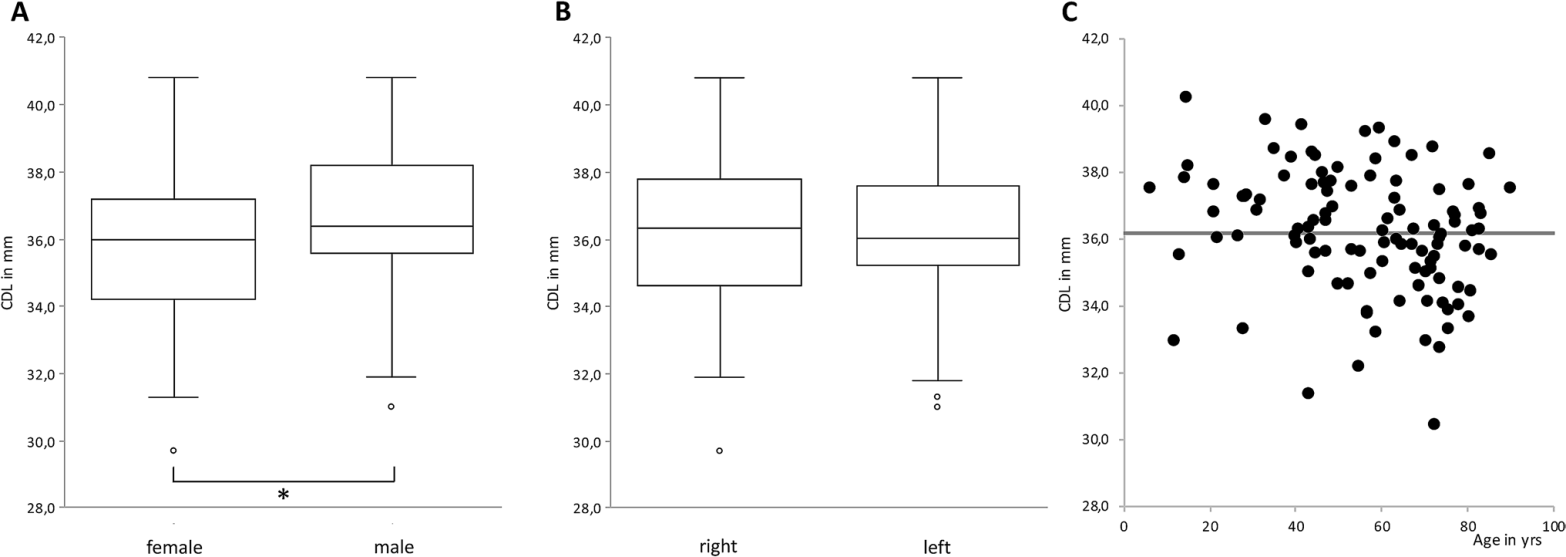
Comparison of cochlear duct length—female versus male, right versus left and age distribution. **A** Comparison of CDL between female and male individuals. The asterisk marks statistically significant differences, the empty circles indicate outliners. **B** Comparison of cochlear duct length between right and left. The empty circles indicate outliners. **C** Age distribution in years of all analyzed individuals versus CDL. The black dots indicate each investigated individual, the grey solid line at 36.2 mm the mean of the whole investigated cohort. *CDL* cochlear duct length

**Fig. 5:**
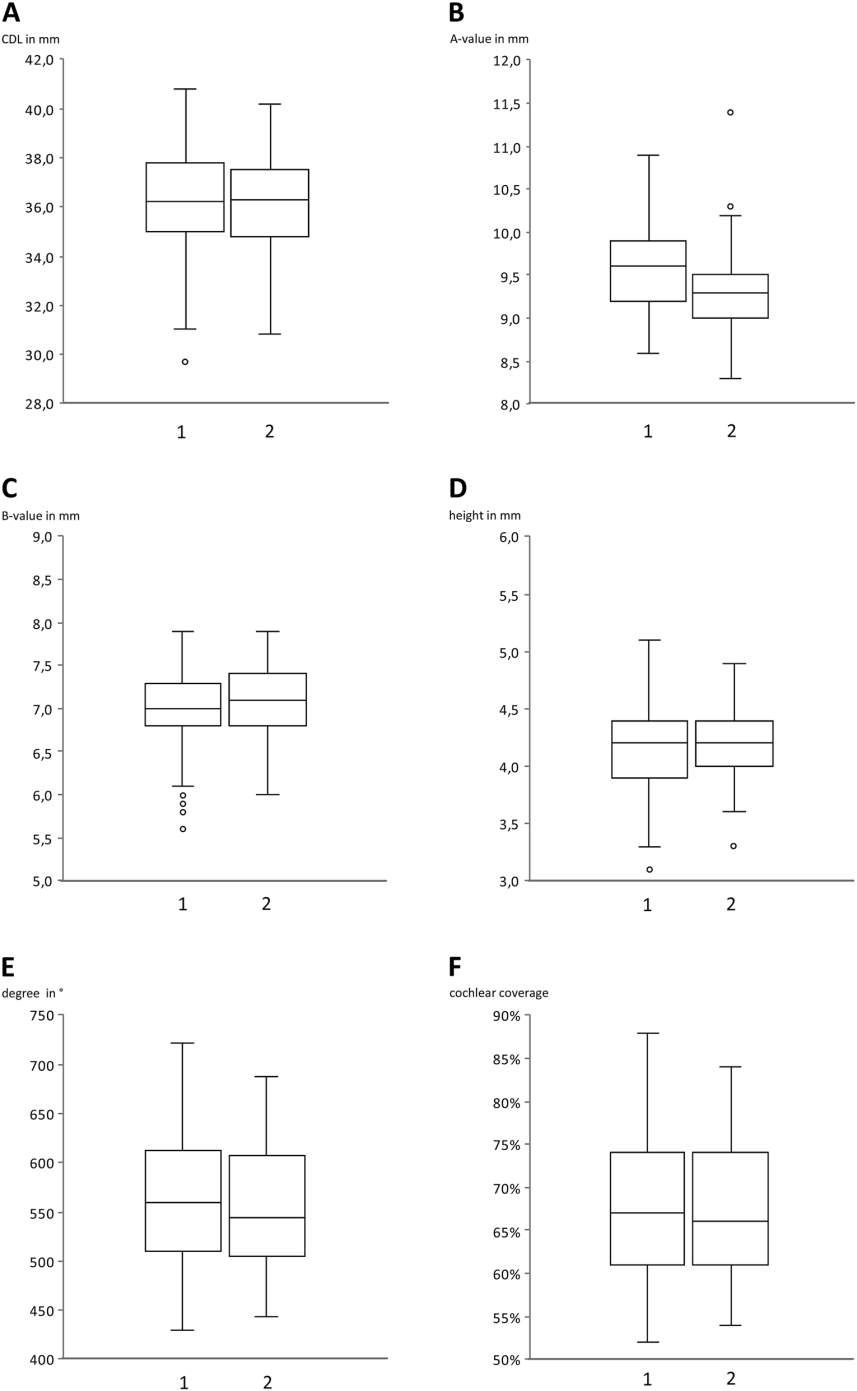
Interrater variability. Interrater reliability between the two blinded investigators. **A** Comparison of CDL (*p *value = 0.887) between investigator 1 (left; 36.2 ± 2.1 mm) and investigator 2 (right; 36.2 ± 1.8 mm). The empty circles indicate outliners. **B** Comparison of A value (*p *value = 0.454) between investigator 1 (left; 9.5 ± 0.5 mm) and investigator 2 (right; 9.3 ± 0.5 mm). The empty circles indicate outliners. **C** Comparison of B value (*p *value = 0.412) between investigator 1 (left; 7.0 ± 0.5 mm) and investigator 2 (right; 7.1 ± 0.4 mm). The empty circles indicate outliners. **D** Comparison of height of the cochlea (*p *value = 0.764) between investigator 1 (left; 4.2 ± 0.4 mm) and investigator 2 (right; 4.2 ± 0.3 mm). The empty circles indicate outliners. **E** Comparison of AID (*p *value = 0.519) between investigator 1 (left; 561.3 ± 63.6°) and investigator 2 (right; 557.4 ± 65.5°). The empty circles indicate outliners. **F** Comparison of cochlear coverage (*p *value = 0.627) between investigator 1 (left; 67.8 ± 8.0%) and investigator 2 (right; 67.9 ± 8.0%). The empty circles indicate outliners. *AID* angular insertion depth; *CDL* cochlear duct length

The mean CDL of the whole investigated cohort was 36.2 ± 1.8 mm (range: 30.4 – 40.2 mm) with a variation over 30%. No significant differences between the groups of different electrode types were observed (FLEX28™: 36.2 ± 2.0 mm; FLEXSOFT™: 36.2 ± 1.4 mm; *p* = 0.922). The mean AID was 528.3 ± 46.3° for the FLEX28™ and 622.2 ± 35.5° for the FLEXSOFT™ group (*p* < 0.001), resulting in a cochlear coverage of 63.9 ± 5.6% for FLEX28™ versus 75.8 ± 4.3% for FLEXSOFT™, respectively. All data regarding the morphology are listed in Table 1. In none of the individuals an AID of 720° was reached, which would equal to two turns of the cochlear. Figure 3A shows the relation of the AID to the CDL with regard to the type of electrode. When looking at a distribution function, half of the examined cochleae are in a range of 35.0 – 37.0 mm (Fig. 3B). In addition, only two cochleae (1.8%) were measured shorter than 32.0 mm, which means that in the rest of the cohort an electrode of 31.0 mm length would have fitted. Comparing the CDL between females (35.8 ± 0.3 mm) and males (36.5 ± 0.2 mm), a significant difference was observed (*p* = 0.037; Fig. 4A). Right (36.1 ± 0.2 mm) and left (36.3 ± 0.3 mm) ears showed no statistically different CDL (*p* = 0.681; Fig. 4B). Concerning a difference in CDL according to the age of the analyzed individuals, we found no difference or trend, as depicted in Fig. 4C. The analysis of the interrater reliability showed no significant differences between the two rater’s measurements (CDL: *p *= 0.887; A value: *p *= 0.454; B value: *p *= 0.412; height of the cochlea: *p *= 0.764; AID: *p *= 0.519; cochlear coverage: *p *= 0.627; Fig. 5 A–F).

**Table 1:** Morphology of the cochleae

Morphology	FLEX 28		FLEX SOFT		*p *value
CDL (mm ± SD)	36.2	2.0	36.2	1.4	0.922
A value (mm ± SD)	9.4	0.4	9.5	0.4	0.559
B value (mm ± SD)	7.0	0.4	7.0	0.3	0.465
Height (mm ± SD)	4.2	0.3	4.2	0.3	0.793
AID ( ± SD)	528.3	46.3	622.2	35.5	< 0.001
Cochlear coverage (% ± SD)	63.9	5.6	75.8	4.3	< 0.001
Length of C1 electrode contact (Hz ± SD)	402.0	103.6	211.5	56.3	< 0.001

*AID* angular insertion depth; *CDL* cochlear duct length; *n* number; *SD* standard deviation

## Discussion

Our analysis showed a significant difference of mean CDL with regard to sex, but not to age, side, or patients having received different types of CI-electrodes. Thus, a very broad and significant range in the CDL was observed with only two patients (1.8%) exhibiting cochleae shorter than 31.0 mm. In all patients the intended AID of approximately 720°, equal to two turns of the cochlea, was not reached, even if standard postoperative radiologic checkup via a Stenvers view head X-ray would suggest.

Other study groups, like Canfarotta and collaborators, have so far investigating the CDL, AID, and cochlear coverage of CI patients with a tablet-based software [19, 24]. Canfarotta’s first study concentrated on validating the technique with the OTOPLAN software on a fairly smaller sample size than our study with analyzing inter- and intrarater reliability and calculating the AID. In comparison, our study focused on the anatomy with the analysis of differences in gender and age. Due to the large data set, our study benefits from an equal share of gender and sides, as well as a broad range of age. A possible downside, we see in the presented cohort, is the relatively small share of younger children, which lacks to examine the growth of the temporal bone in early age; however, cochlear size is known to be at the same size at birth and in adult age [25]. Validation of the re-test variability of the OTOPLAN software was also performed in our study by calculating the excellent interrater reliability. The second study of Canfarotta et al. investigated the variability in frequency-to-place mismatch in 111 recipients listening with CI and electric-acoustic stimulation (EAS) measuring the respective AID with the OTOPLAN software with lesser focus on anatomic variations [24]. Other studies analyzing the CDL regarding age or sex had smaller cohorts and lacked of an analysis of the interrater reliability [26, 27].

When comparing the presented values of CDL to studies using different techniques (Table 2), predominantly similar values are observed. Comparing the CDL with studies using the direct technique, a method where the CDL is measured directly on histologic slices, similar values to our study are found. In the largest cohort of 50 individuals applying the direct technique, Ulehlova and collaborators found a similar mean to our study of 34.2 mm with a similar range of 28.0 – 40.1 mm [4]. Other studies with smaller numbers (*n* = 5 to 35) observed lower means of CDL (33.13 – 34.0 mm) [28–31]. The comparison to studies generating the CDL with the 3D-reconstruction method showed similar mean values and ranges [14, 32–35] in very large cohorts up to 436 individuals [34], as well. A predecessor technique to the method used with the OTOPLAN software is the technique of the spiral coefficient, introduced by Alexiades and collaborators [17]. Studies using this technique calculated similar values [15, 36–38]. Looking at studies using the OTOPLAN software, mean of CDL was lower (32.4 – 34.0 mm; Table 2) Comparing the AID of the study of Canfarotta and collaborators [19], slightly lower values were observed in our cohort with regard to the AID (525.4° versus 578°) for FLEX28 patients, and similar values for FLEXSOFT patients (615.4° versus 619°). The application of the indirect technique, where a stack of histologic slices is used to reconstruct a two-dimensional model of the cochlea showed a broader variation in the mean CDL (range of mean: 28.4–42.0 mm) [3, 39–43], which is accompanied by a known method error effect depending on the cutting angle of the histologic slices [33].

**Table 2:** Variation of cochlear duct length with regard to the different measuring techniques^a^

Author	Year	*n*	Modality	Slice thickness	Mean of CDL (SD) in mm	Range of values in mm	Calculated difference in %
Direct technique							
Retzius [3]	1884	5	Histology	n/a	33.5 (0.8)	32.0–34.0	6.3
Bredberg [28]	1968	35	Histology	n/a	31.5 (2.3)	30.3–37.6	24.1
Úlehlová et al. [4]	1987	50	Histology	n/a	34.2 (2.9)	28.0–40.1	43.2
Wright et al. [31]	1987	14	Histology	n/a	32.9 (2.6)	28.8–36.6	27.1
Sridhar et al. [29]	2006	7	Histology	n/a	33.3 (2.4)	30.5–36.9	20.1
Stakhovskaya et al. [30]	2007	9	Histology	n/a	33.1 (2.1)	30.5–36.9	20.1
Indirect technique							
Hardy [3]	1938	68	Histology	n/a	31.5 (2.3)	25.6–35.5	38.7
Walby et al. [41]	1985	20	Histology	n/a	32.6 (2.1)	30.1–36.6	21.6
Pollak et al. [40]	1987	9	Histology	n/a	28.4 (3.4)	24.0–33.5	39.6
Erixon et al. [39]	2009	58	Plastic casts	n/a	42.0 (2.0)	38.6–45.6	18.1
Lee et al. [42]	2010	27	Histology	n/a	30.8 (2.6)	25.5–35.1	37.6
Erixon and Rask-Andersen [43]	2013	51	Plastic casts	n/a	41.2 (1.9)	37.6–44.9	16.3
3D reconstruction							
Takagi and Sando [14]	1989	1	Histology	n/a	36.4 (n/a)	n/a	n/a
Sato et al. [32]	1991	18	Histology	n/a	34.7 (2.9)	29.7–38.9	31.0
Kawano et al. [33]	1996	8	Histology	n/a	35.6 (1.4)	34.2–37.9	10.8
		8	Histology	n/a	40.8 (2.0)	37.9–43.8	15.6
Würfel et al. [34]	2014	218	In vivo CBCT	0.3 mm voxel	37.9 (2.0)	30.8–43.2	40.3
Meng et al. [35]	2016	310	In vivo CT	0.7 mm	35.8 (2.0)	30.7–42.2	37.5
Spiral coefficients							
Ketten et al. [15]	1998	20	In vivo CT	1.0 mm	33.0 (2.3)	29.1–37.5	28.9
Skinner et al. [36]	2002	26	In vivo CT	1.0 mm	34.6 (1.2)	32.9–36.6	11.2
Alanazi and Alzhrani [47]	2018	401	In vivo CT	< 1.0 mm	31.9 (n/g)	20.3–37.7	85.7
Grover et al. [38]	2018	124	In vivo CT	< 1.0 mm	29.8 (n/g)	28.0–34.3	22.5
Tablet-based software							
Canfarotta et al. [19]	2019	20	In vivo CT	n/g	n/g, only AID	n/g, only AID	n/a
Canfarotta et al. [24]	2020	111	In vivo CT	n/g	34.0 (1.9)	29.4–39.5	34.4
Lovato et al. [26]	2020	5	In vivo CT	n/g	32.4 (n/g)	30.7–33.3	8.0
Khurayzi et al. [27]	2020	88	In vivo CT	n/g	32.9 (1.8)	28.1–37.8	27.4
Present study	2021	108	In vivo CT	< 0.7 mm	36.2 (1.8)		32.2tab

^a^Modified according to Koch RW, et al. [11]

*AID* angular insertion depth; *CDL* cochlear duct length; *n/a* not applicable; *n/g* not given; *SD* standard deviation

Concerning the observed significant difference of CDL in male and female patients in the present study, other studies also found longer cochleae in male individuals [27, 44, 45]. Contributing to the difference might be correlation to a larger height in the individual or a larger head diameter, which has not been investigated so far.

Looking at CDL and AID values generated by the present investigated tablet-based software OTOPLAN are comparable to studies with the named different techniques. In addition, the re-test variability was shown to be very low in both our study and Canfarotta et al. [19]. With a straightforward applicability of this tablet-based software, individualized CI implantation with precise personalized preoperative planning is feasible. With the results of our study, the known variance of the cochlea is once again demonstrated. Even if the morphology seems normal at first glance in the computed tomography, pitfalls might occur intraoperatively, like incomplete insertion in patients with shorter cochlea with kinking or tipfoldover of the electrode, or damage of the lamina spiralis. Interestingly, two cochleae were measured shorter than 32.0 mm, meaning that in the remaining 106 patients the insertion of an electrode of 31.0 mm length, would have been feasible. Moreover, with regard to further implications and improvements of CI, the morphology, CDL and AID play an essential role. Referring to the data of the present study and Canfarotta et al. [19], in none of the FLEXSOFT™ patients the preferred AID of 720°, which would equal to two turns of the cochlea, was reached. This opens the discussion, if CI patients would benefit from longer electrodes covering a broader range of frequencies inside the cochlea. As the group around Canfarotta reported, patients with CI alone and longer electrodes seem to have a lower degree of frequency-to-place mismatch than patients with residual hearing and electric acoustic stimulation [24]. In addition, they found a better long-term speech recognition in patients with a 31.5 mm electrode, than those with a 24 mm array [46]. At the same time, the same research group observed a greater likelihood of preservation of the lower frequencies in patients with longer CDL (up to 36.5 mm) with the same electrode (31.5 mm) [47]. Another study group observed a beneficial influence of deep insertion with regard to thresholds up to 65 dB at 0.5 kHz [48]. Regarding musical sound quality discrimination, cochlea implant users with a 31.5 mm electrode had due to the greater apical stimulation an improved musical low-frequency perception in comparison to those with a 24.0 mm electrode [49]. Whereas, another study group observed no audiological benefit between groups of different electrode lengths (active lengths: 15.0 mm versus 19.1 mm versus 23.1 mm) in single sided deafness patients [50].

Since we know of a certain variation of the CDL in the presented cohort of almost 10 mm, the debate for longer electrodes versus an actual and verifiable audiological benefit in everyday life remains open. Nevertheless, hearing outcome with a cochlear implant is influenced by many other factors, like uni- versus bilateral implantation, psychological state, motivation, socioeconomic status, profession, environment of the patient, etiology of hearing loss, hearing rehabilitation, age and time of implantation that there might not be an answer to the grade of contribution the electrode length has to the hearing result [51–54]. With an international multicenter CI-registry all-encompassing investigations could be generated to potentially answer this question.

## Conclusion

Analysis with the tablet-based software OTOPLAN showed a broad range of CDL with a variation over 30% and significant differences in sex, but none in age or side. This broad range in CDL should be considered preoperatively for issues like avoidance of complications (incomplete insertion, kinking or tipfoldover of the electrode), attempt of atraumatic insertion, individual necessities (hearing preservation, cochlear malformation), and tonotopic matching of electrical stimulation site Further studies with correlation of CDL, hearing results, and tonotopic matching are required for different patient groups. Interestingly, the AID was smaller than Stenvers view head X-ray would have suggested, which again leaves room for the debate about longer electrodes versus a significant audiological benefit for patients in their daily life.

## Supplementary Information

Below is the link to the electronic supplementary material.

Supplementary file1 (PDF 159 KB)

Supplementary file2 (PDF 153 KB)

## Data Availability

Original data is available on demand.

## Code availability

Statistical analysis was performed with the software Real Statistics Resource Pack software (Release 6.8; www.real-statistics.com) and Microsoft^®^ Excel^®^ 16.0.12730.20144. The respective codes are available on demand.
