# Supplementary material for: Variation of the cochlear anatomy and cochlea duct length: analysis with a new tablet-based software
Source: Eur Arch Otorhinolaryngol. 2021 May 29;279(4):1851–61. doi: 10.1007/s00405-021-06889-0 (PMC8930796; doi:10.1007/s00405-021-06889-0)
Supplement: Supplementary file 1 — Supplementary file1 (PDF 159 KB) [file 405_2021_6889_MOESM1_ESM.pdf]

LMU KLINIKUM
Klinik und Poliklinik
für Hals-Nasen-Ohren-Heilkunde
Campus Großhadern
Campus Innenstadt

HNO LMU

LMU Klinikum, Department of Otorhinolaryngology, Ludwig-Maximilians-University Munich
Marchioninistr. 15, 81377 Munich, Germany

Resident for Otorhinolaryngology
Dr. med. Jennifer Spiegel

Tel. +49 89 4400-0
Fax +49 89 4400-76869
Jennifer.Spiegel@
med.uni-muenchen.de
www.lmu-klinikum.de

Prof. Dr. med. Roland Laszig
Editor-in-Chief
European Archives of Oto-Rhino-Laryngology

Revision of the Manuscript "Variation of the Cochlear Anatomy and Cochlea Duct Length: Analysis with a New Tablet-Based Software" **May 11th, 2021**

Manuscript Number: EAOR-D-21-00111

Dear Professor Laszig,

Thank you very much for the opportunity to revise our above-named manuscript. In the following you will find a point-to-point response to the reviewer comments including modifications, that were made to the original manuscript. As well, the modified manuscript is uploaded to the website with yellow-highlighted changes.

Thank you again and with best regards,

Jennifer Spiegel
(on behalf of the author team)

Vorstand

Ärztlicher Direktor:
Prof. Dr. med. Markus M. Lerch
(Vorsitz)

Kaufmännischer Direktor:
Markus Zendler

Pflegedirektor:
Marcus Huppertz

Vertreter der Medizinischen Fakultät:
Prof. Dr. med. dent. Reinhard Hickel
(Dekan)

Institutionskennzeichen:
260 914 050

Umsatzsteuer-ID:
DE813536017

Das Klinikum der Universität
München ist eine Anstalt des
Öffentlichen Rechts

COMMENTS TO THE AUTHOR:

Reviewer #1:

With the article entitled "Variation of the Cochlear Anatomy and Cochlea Duct Length: Analysis with a New Tablet-Based Software", authors described their experience in calculating retrospectively the cochlear duct length through a new software.

Although the paper is well written, the message and the interest delivered by the paper is poor. There is still no evidence that a deeper insertion with a supposed better tonotopy gives better audiological results. The interest of measuring the cochlear length is still not evident and could sound as an author preference for choosing a specific cochlear implant without any evident benefit for the patient

We thank Reviewer #1 for the time reviewing our paper and want to clarify the message of our paper.
This paper depicts descriptively the large range of CDL over almost 1 cm (30.4 – 40.2 mm) in the evaluated cohort. Indeed, the impact of cochlear coverage, tonotopy, and atraumatic insertion on audiological results is widely debated [1]. Nevertheless, hearing outcome with a cochlear implant is influenced by so many diverse factors, ranging from the uni- versus bilateral implantation, psychological state, motivation, socioeconomic status, profession, environment of the patient, etiology of hearing loss, hearing rehabilitation, age and time of implantation [2-4], etc., to potentially also the choice of the electrode length. In cases with shorter cochlea or inner ear malformation [5], residual hearing [6], or prevention of vertigo [7] considering the length of the electrode is crucial. The influence of cochlear coverage in patients with a “normal” anatomy is still under evaluation [8-15] and may be without a final answer. Several study groups report of a certain audiological relevance of stimulating the apical region [9-14]. Our data merely depicts the wide individual range concentrating on the anatomy of the cochlea without delivering hearing results. In a next step it would be interesting to further investigate the correlation of the cochlear coverage with hearing results for different patient groups separately, e.g. single sided deafness, groups with different time of onset of deafness, socioeconomic status, educational status, age, etc. With our work, we also wanted to point out, that the favored “2 turns of the cochlea” (=720°) for a “complete insertion” were not reached in any of the investigated patients. This brings us back to the discussion of how long cochlear implant electrodes for a “complete insertion” should be, or how many degrees of angular insertion depth for electric stimulation would be required for a recognizable benefit in the patient’s everyday life.

References:

[1] An overview of cochlear implant electrode array designs. Dhanasingh A, Jolly C. Hear Res. 2017 Dec;356:93-103. doi: 10.1016/j.heares.2017.10.005

[2] Blamey P, Artieres F, Baskent D, Bergeron F, Beynon A, Burke E, Dillier N, Dowell R, Fraysse B, Gallego S, Govaerts PJ, Green K, Huber AM, Kleine-Punte A, Maat B, Marx M, Mawman D, Mosnier I, O'Connor AF, O'Leary S, Rousset A, Schauwers K, Skarzynski H, Skarzynski PH, Sterkers O, Terranti A, Truy E, Van de Heyning P, Venail F, Vincent C, Lazard DS (2013) Factors affecting auditory performance of postlinguistically deaf adults using cochlear implants: an update with 2251 patients. Audiol Neurootol 18 (1):36-47. doi:10.1159/000343189

[3] Lazard DS, Vincent C, Venail F, Van de Heyning P, Truy E, Sterkers O, Skarzynski PH, Skarzynski H, Schauwers K, O'Leary S, Mawman D, Maat B, Kleine-Punte A, Huber AM, Green K, Govaerts PJ, Fraysse B, Dowell R, Dillier N, Burke E, Beynon A, Bergeron F, Baskent D, Artieres F, Blamey PJ (2012) Pre-, per- and postoperative factors affecting performance of postlinguistically deaf adults using cochlear implants: a new conceptual model over time. PLoS One 7 (11):e48739. doi:10.1371/journal.pone.0048739

[4] Tyler RS, Moore BC, Kuk FK (1989) Performance of some of the better cochlear-implant patients. J Speech Hear Res 32 (4):887-911. doi:10.1044/jshr.3204.887

[5] Geier L, Barker M, Fisher L, Opie J (1999) The effect of long-term deafness on speech recognition in postlingually deafened adult CLARION cochlear implant users. Ann Otol Rhinol Laryngol Suppl 177:80-83. doi:10.1177/00034894991080s416

[6] Sennaroglu L, Sarac S, Ergin T (2006) Surgical results of cochlear implantation in malformed cochlea. Otol Neurotol 27 (5):615-623

[7] Bruce I, Todt I (2018) Hearing Preservation Cochlear Implant Surgery. Adv Otorhinolaryngol 81:66-73. doi:10.1159/000485544

[8] Nordfalk K, Rasmussen K, Hopp E, Bunne M, Silvola J, Jablonski G (2016) Insertion Depth in Cochlear Implantation and Outcome in Residual Hearing and Vestibular Function. Ear Hear 37 (2):e129-e137. doi:10.1097/AUD.0000000000000241

[9] Hochmair I, Arnold W, Nopp P, Jolly C, Müller J, Roland P (2003). Deep electrode insertion in cochlear implants: apical morphology, electrodes and speech perception results. Acta Otolaryngol 123 (5):612-7

[10] Canfarotta MW, Dillon MT, Buss E, Pillsbury HC, Brown KD, O'Connell BP (2020) Frequency-to-Place Mismatch: Characterizing Variability and the Influence on Speech Perception Outcomes in Cochlear Implant Recipients. Ear Hear 41 (5):1349-1361. doi:10.1097/AUD.0000000000000864

[11] Hollis ES, Canfarotta MW, Dillon MT, Rooth MA, Bucker AL, Dillon SA, Young A, Quinones K, Pillsbury HC, Dedmon MM, O'Connell BP, Brown KD (2021) Initial Hearing Preservation Is Correlated With Cochlear Duct Length in Fully-inserted Long Flexible Lateral Wall Arrays. Otol Neurotol. doi:10.1097/MAO.0000000000003181

[12] Helbig S, Adel Y, Leinung M, Stover T, Baumann U, Weissgerber T (2018) Hearing Preservation Outcomes After Cochlear Implantation Depending on the Angle of Insertion: Indication for Electric or Electric-Acoustic Stimulation. Otol Neurotol 39 (7):834-841. doi:10.1097/MAO.0000000000001862

[13] Roy AT, Penninger RT, Pearl MS, Wuerfel W, Jiradejvong P, Carver C, Buechner A, Limb CJ (2016) Deeper Cochlear Implant Electrode Insertion Angle Improves Detection of Musical Sound Quality Deterioration Related to Bass Frequency Removal. Otol Neurotol 37 (2):146-151. doi:10.1097/MAO.0000000000000932

[14] Canfarotta MW, Dillon MT, Buchman CA, Buss E, O'Connell BP, Rooth MA, King ER, Pillsbury HC, Adunka OF, Brown KD (2021) Long-Term Influence of Electrode Array Length on Speech Recognition in Cochlear Implant Users. Laryngoscope 131(4):892-897. doi: 10.1002/lary.28949

[15] Speck I, Ketterer MC, Arndt S, Aschendorff A, Jakob TF, Hassepass F (2021) Comparison of Speech Recognition and Localization Ability in Single-sided Deaf Patients Implanted With Different Cochlear Implant Electrode Array Designs. Otol Neurotol 42 (1):e22-e32. doi:10.1097/MAO.0000000000002864

Accordingly, we have changed the following paragraph in the discussion part, conclusion, and the conclusion part of the abstract (References, please see manuscript):

<u>Abstract/Conclusion</u> (page 1): "A broad range of CDL was observed with significant larger values in male, but no significant differences concerning side or age. Almost every cochlea was measured longer than 31.0 mm. Preoperative assessment aids in prevention of complications (incomplete insertion, kinking, tipfoldover), attempt of atraumatic insertion, and addressing individual necessities (hearing preservation, cochlear malformation). The preferred AID of 720° (two turns of the cochlea) was never reached, opening the discussion for the requirement of longer CI-electrodes versus a debatable audiological benefit for the patient in his/her everyday life."

<u>Discussion</u> (page 10-11): "This opens the discussion, if CI patients would benefit from longer electrodes covering a broader range of frequencies inside the cochlea. As the group around Canfarotta reported, patients with CI alone and longer electrodes seem to have a lower degree of frequency-to-place mismatch than patients with residual hearing and electric acoustic stimulation [24]. In addition, they found a better long-term speech recognition in patients with a 31.5 mm electrode, than those with a 24 mm array [46]. At the same time, the same research group observed a greater likelihood of preservation of the lower frequencies in patients with longer CDL (up to 36.5 mm) with the same electrode (31.5 mm) [47]. Another study group observed a beneficial influence of deep insertion with regard to thresholds up to 65 dB at 0.5 kHz [48]. Regarding musical sound quality discrimination, cochlea implant users with a 31.5 mm electrode had due to the greater apical stimulation an improved musical low-frequency perception in comparison to those with a 24.0 mm electrode [49]. Whereas, another study group observed no audiological benefit between groups of different electrode lengths (active lengths: 15.0 mm versus 19.1 mm versus 23.1 mm) in single sided deafness patients [50].
Since we know of a certain variation of the CDL in the presented cohort of almost 10 mm, the debate for longer electrodes versus an actual and verifiable audiological benefit in everyday life remains open. Nevertheless, hearing outcome with a cochlear implant is influenced by many other factors, like uni- versus bilateral implantation, psychological state, motivation, socioeconomic status, profession, environment of the patient, etiology of hearing loss, hearing rehabilitation, age and time of implantation that there might not be an answer to the grade of contribution the electrode length has to the hearing result [51-54]. With an international multicenter CI-registry all-encompassing investigations could be generated to potentially answer this question."

<u>Conclusion</u> (page 12): "Analysis with the tablet-based software OTOPLAN showed a broad range of CDL with a variation over 30% and significant differences in sex, but none in age or side. This broad range in CDL should be considered preoperatively for issues like avoidance of complications (incomplete insertion, kinking or tipfoldover of the electrode), attempt of atraumatic insertion, individual necessities (hearing preservation, cochlear malformation), and tonotopic matching of electrical stimulation site Further studies with correlation of CDL,

hearing results, and tonotopic matching are required for different patient groupsInterestingly, the AID was smaller than Stenvers view head x-ray would have suggested, which again leaves room for the debate about longer electrodes versus a significant audiological benefit for patients in their daily life.”

Reviewer #2:

Well written several comments:

(1) *In the introduction you wrote: "Nowadays, thorough preoperative planning with measurement of the CDL assists in choosing the correct electrode length". It is important to explain why? For example - Are some cochleas smaller and need shorter electrodes?*

Ad (1):

Thank you for taking your time reviewing our paper. Since the impressive work of Erixon *et al*. [1], we know of the extensive variation of cochlear anatomy. We indeed observed that the range in cochlear duct length (CDL) varies around 1 cm (30.4 – 40.2 mm). In a cochlea with 30.4 mm length, the surgeon would not be able to insert a 31 mm long electrode completely. Depending on different individual necessities of the patient (hearing preservation, inner ear malformations, etc.) with considering the CDL, the electrode should be chosen accordingly.

Reference:

[1] Erixon E, Högstorp H, Wadin K, Rask-Andersen H (2009). Variational anatomy of the human cochlea: implications for cochlear implantation. Otol Neurotol 30 (1):14-22. doi: 10.1097/MAO.0b013e31818a08e8.

(2) *At the end of the introduction please write the objectives of the study.*

Ad (2):

As recommended, we added the objectives of the study in the introduction part as follows (page 4):
“Objectives of the study were to evaluate the range of CDL, find differences in different patient groups (sex, age, type of electrode), and to assess the angular insertion depth (AID) for the cochlear coverage.”

(3) *Methods "Patients who received a FLEX 28 electrode were implanted with the aim of hearing preservation" This sentence is very strange and needs explanation. Implants that preserve hearing are traditionally up to 24 mm.*

Ad (3):

We agree that traditionally a 20 to 24 mm electrode would be an option to achieve hearing preservation with the aim of electric acoustic stimulation. Nevertheless, the choice for the length of the electrode is influenced by individual anatomy, residual hearing, and the philosophy of the surgeon. The analyzed cohort of Flex 28 patients have all been implanted by the same surgeon with either the aim of prevention of vertigo or hearing preservation in patients with minimal residual hearing. In all of those patients the residual hearing was within the lower frequencies, however beyond the indication range for electrical acoustic stimulation. If the reviewer feels this sentence is irrelevant for the conclusion of the study, since the study merely focusses on the evaluated anatomic data and not on the hearing outcome, we will omit this sentence.

(4) *In Data analysis: "a slice thickness ⩾ 0.7 mm were excluded from the study" this is redundant as it was stated earlier in Methods*

Ad (4):

We omitted the following sentence (page 6):

"CT scans of patients suffering from inner ear anomalies and/or with a slice thickness ≥ 0.7 mm were excluded from the study."

(5) *Results: "Due to a high rate of scans with slice thickness of 0.7 mm or more, 72 FLEX28 implanted ears and 36 FLEXSOFT implanted ears were included into the investigation (Figure 1)." You mean "were excluded"*

Ad (5):

Thank you for pointing out the unclear phrasing of the sentence. In order to clarify, we have rephrased it (page 8): "Due to a high rate of scans with slice thickness of 0.7 mm or more, only a total of 72 FLEX28 implanted ears and 36 FLEXSOFT implanted ears were included into the investigation (Figure 1)."

(6) *Fig. 1 3rd rectangle "118 CT-scan data sets with adequate slight thickness (< 0.6mm) (80 Flex 28, 38 Flex soft)" should be < 0.7mm or ⩽ 0.6mm*

Ad (6):

We have changed it to "⩽ 0.6mm" in Figure 1

(7) *I would think of another implication of the study. We perform medical tests in order to make better decisions. The data you present that it is very unusual that the length of the cochlear is less than 31 mm does not support performing preoperative measurements as doesn't really affect the choice of what length of electrode should be used. Please discuss why you think that preop measurements are required.*

Ad (7):

When looking into the literature using different or even the same measuring technique, a broad range of CDL is reported (see Table 2). In addition, a number of studies report of individuals with shorter CDL than 31.0 mm. Thus, preoperative measuring of the CDL contributes to the surgical management, in particular, when the cochlea seems smaller/shorter/flatter in the CT scan at first glance. Without getting familiar with the surgical anatomy preoperatively, intraoperative complications like trying to insert a 31.0 mm long electrode into a smaller cochlea which could damage the lamina spiralis or result in incomplete insertion with kinking or tipfoldover of the electrode.

We changed the following paragraph to the paper (discussion and conclusion):

Discussion (page 10): "Even if the morphology seems normal at first glance in the computed tomography, pitfalls might occur intraoperatively, like incomplete insertion in patients with shorter cochlea with kinking or tipfoldover

of the electrode, or damage of the lamina spiralis. Interestingly, two cochleae were measured shorter than 32.0 mm, meaning that in the remaining 106 patients the insertion of an electrode of 31.0 mm length, would have been feasible. Moreover, with regard to further implications and improvements of CI, the morphology, CDL and AID play an essential role."

Conclusion (page 12): "Analysis with the tablet-based software OTOPLAN showed a broad range of CDL with a variation over 30% and significant differences in sex, but none in age or side. This broad range in CDL should be considered preoperatively for issues like avoidance of complications (incomplete insertion, kinking or tipfoldover of the electrode), attempt of atraumatic insertion, individual necessities (hearing preservation, cochlear malformation), and tonotopic matching of electrical stimulation site."
