# Supplementary material for: Variation of the cochlear anatomy and cochlea duct length: analysis with a new tablet-based software
Source: Eur Arch Otorhinolaryngol. 2021 May 29;279(4):1851–61. doi: 10.1007/s00405-021-06889-0 (PMC8930796; doi:10.1007/s00405-021-06889-0)
Supplement: Supplementary file 2 — Supplementary file2 (PDF 153 KB) [file 405_2021_6889_MOESM2_ESM.pdf]

Department of Otorhinolaryngology
LMU Klinikum, Ludwig-Maximilians-Universität München, Munich, Germany

Department of Otorhinolaryngology
Chair: Prof. Dr. Martin Canis

Manuel Bernal-Sprekelsen

Department of Otorhinolaryngology—Head and Neck Surgery at the Hospital Clinic, University of Barcelona, Spain

Dr. med. Jennifer L. Spiegel
Resident Otorhinolaryngology

Marchioninistr. 15, 81377 Muenchen, Germany **Postal Address**
+49 89 / 4400-0 **Telefone**
+49 89 / 4400-76869 **Facsimile**
jennifer.spiegel@med.uni-muenchen.de **E-Mail**

Editor-in-Chief
European Archives of Oto-Rhino-Laryngology

January 15, 2021

Submission of a manuscript titled: “**Variation of the Cochlear Anatomy and Cochlear Duct Length: Analysis with a New Tablet-Based Software**”

Dear Professor Bernal-Sprekelsen,

enclosed, please find the submission to our results on an investigation of the variation of the cochlea and cochlear duct length in respect to an analysis with a tablet-based software.

In cochlear implantation, thorough preoperative planning together with measurement of the cochlear duct length (CDL) assists in choosing the right electrode. For measuring the CDL, different techniques have been introduced in the past century along with the at the time available technology. A tablet-based software offers an easy and intuitive way to visualize and analyze the anatomy of the temporal bone, its proportions and measure the CDL. Therefore, we investigated the calculation technique of the CDL via a tablet-based software on our own cohort retrospectively. We found a broad range of CDL with a variation of over 30%, and significant differences in sex, but none in age or side. Almost every cochlea (98%) was measured longer than 31.0 mm. Therefore, if a tonotopic matching of electrical stimulation site with variations of cochlea anatomic length is intended, it requires a set of different electrode lengths to choose accordingly. Interestingly, the AID was smaller than Stenvers view head x-ray would have suggested.

We hope that our manuscript meets the requirements and the high standards of your journal *European Archives of Oto-Rhino-Laryngology* in order to be considered for publication.

Best regards,

Jennifer Spiegel

on behalf of the author team
